# Quantitation of the neural silencing activity of anion channelrhodopsins in *Caenorhabditis elegans* and their applicability for long-term illumination

**DOI:** 10.1038/s41598-019-44308-x

**Published:** 2019-05-27

**Authors:** Taro Yamanashi, Misayo Maki, Keiichi Kojima, Atsushi Shibukawa, Takashi Tsukamoto, Srikanta Chowdhury, Akihiro Yamanaka, Shin Takagi, Yuki Sudo

**Affiliations:** 1 Graduate School of Medicine, Dentistry and Pharmaceutical Sciences, Okayama University, Okayama, 700-8530 Japan; 2 Department of Neuroscience II, Research Institute of Environmental Medicine, Nagoya University, Nagoya, 464-8601 Japan; 3 Division of Biological Science, Graduate School of Science, Nagoya University, Nagoya, 464-8602 Japan; 4 Present Address: Faculty of Advanced Life Science and Global Station for Soft Matter, Global Institution for Collaborative Research and Education, Hokkaido University, Kita-10 Nishi-8, Kita-ku, Sapporo, 060-0810 Japan

**Keywords:** Membrane biophysics, Molecular biophysics

## Abstract

Ion pumps and channels are responsible for a wide variety of biological functions. Ion pumps transport only one ion during each stimulus-dependent reaction cycle, whereas ion channels conduct a large number of ions during each cycle. Ion pumping rhodopsins such as archaerhodopsin-3 (Arch) are often utilized as light-dependent neural silencers in animals, but they require a high-density light illumination of around 1 mW/mm^2^. Recently, anion channelrhodopsins -1 and -2 (GtACR1 and GtACR2) were discovered as light-gated anion channels from the cryptophyte algae *Guillardia theta*. GtACRs are therefore expected to silence neural activity much more efficiently than Arch. In this study, we successfully expressed GtACRs in neurons of the nematode *Caenorhabditis elegans* (*C*. *elegans*) and quantitatively evaluated how potently GtACRs can silence neurons in freely moving *C*. *elegans*. The results showed that the light intensity required for GtACRs to cause locomotion paralysis was around 1 µW/mm^2^, which is three orders of magnitude smaller than the light intensity required for Arch. As attractive features, GtACRs are less harmfulness to worms and allow stable neural silencing effects under long-term illumination. Our findings thus demonstrate that GtACRs possess a hypersensitive neural silencing activity in *C*. *elegans* and are promising tools for long-term neural silencing.

## Introduction

In environments on the earth, there are a variety of ions and organisms have ion transporters on their cell membranes that transport ions such as H^+^, Na^+^, K^+^ and Cl^−^. Ion transporters are roughly divided into two types: (i) ion pumps that actively transport ions across the membrane against ion concentration gradient, and (ii) ion channels that conduct ions across the membrane according to the ion concentration gradient^1^. The exchange of ions between the exterior and interior of cells is achieved by the function of pumps and channels, which lead to the production and dissipation of membrane potentials in living organisms^1^. The membrane potential plays crucial roles in the control of biological activities. For instance, an H^+^ pump in mitochondria produces an H^+^ gradient across the cell membrane that is necessary for the synthesis of adenosine triphosphate (ATP)^2^, while the influx of Na^+^ into a cell by a Na^+^ channel triggers the action potential required for cell-to-cell communication, especially neurotransmission^3^.

**Figure 1:**
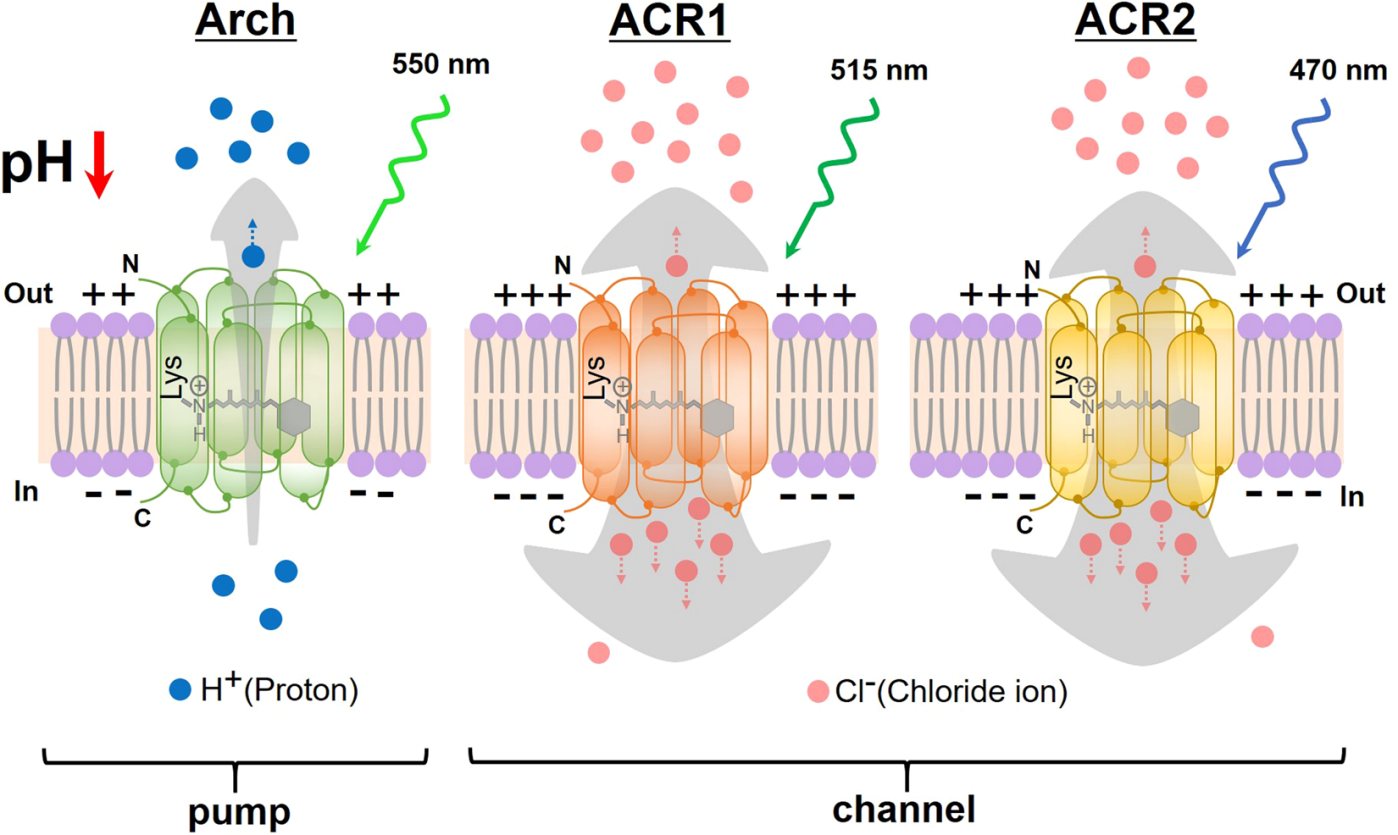
Schematic representations of Arch, GtACR1 and GtACR2. The proton pump Arch actively transports one H^+^ during each photocycle against the H^+^ gradient from the inside to the outside, resulting in a decrease of the outside pH. The H^+^ transport also causes membrane potentials to be more negative and this hyperpolarization silences neurons. In contrast, anion channelrhodopsins, GtACR1 and GtACR2, conduct Cl^−^ ions during each photocycle according to the Cl^−^ gradient from the outside to the inside. GtACRs are therefore capable of efficiently inducing hyperpolarization. “N” and “C” indicate the N- and C-terminus, respectively. Arch, GtACR1 and GtACR2 absorb light at 550 nm, 515 nm and 470 nm, respectively.

Rhodopsins are photoreceptor proteins that possess a chromophore retinal (vitamin-A aldehyde) inside a seven-transmembrane α-helical apoprotein, opsin^4^, in which the retinal forms a Schiff base linkage with a conserved Lys residue (Fig. 1). Rhodopsins are roughly classified into two groups, microbial rhodopsins (type-1) and animal rhodopsins (type-2). Recently, a novel group named heliorhodopsins was identified by Béjà and coworkers^5^, although their biological function is still unclear. Type-1 microbial rhodopsins are widely distributed in a variety of microorganisms including archaea, bacteria and eukaryotes (*i*.*e*., fungi and algae)^6^. The photoabsorption of type-1 microbial rhodopsins triggers the *trans-cis* photoisomerization of the retinal chromophore, and then, through various distinct photointermediates, it thermally returns to the initial state^4^. During this sequential photoreaction called the photocycle, the opsin structurally changes, leading to various biological functions such as ion transport and light sensing^7–9^. When ion pumping and ion channeling microbial rhodopsins are expressed in cells, the production and dissipation of membrane potentials can be artificially regulated by light.

The method named optogenetics enables the manipulation of neural activity with a high spatiotemporal resolution and has been successfully applied to living animals including mice, zebrafish and the nematode *Caenorhabditis elegans* (*C*. *elegans*)^9–14^. The neural inhibition can be evoked through hyperpolarization of the membrane potential and light-driven outward proton pumping rhodopsins such as archaerhodopsin-3 (Arch) and thermophilic rhodopsin (TR) have been used for neural inhibition in animals^14–16^. A light-driven inward chloride pumping rhodopsin, *Natronomonas pharaonis* halorhodopsin (*Np*HR), can be also applied for neural inhibition^17^. However, unlike ion channeling rhodopsins such as light-gated cation channelrhodopsins (CCRs)^9^, ion pumping rhodopsins transport only one ion during each photocycle, which intrinsically limits the number of transportable ions, resulting in a poor efficiency of the hyperpolarization response (Fig. 1). In addition, the movement of a proton (H^+^) across the cell membrane by Arch decreases the extracellular pH, and therefore undesired secondary effects can be induced (Fig. 1). In 2015, light-gated anion channelrhodopsins -1 and -2 (GtACR1 and GtACR2) were discovered in the cryptophyte algae *Guillardia theta*^18^. From electrophysiological analysis of mammalian cells in culture, it has been reported that GtACRs selectively conduct monovalent anions such as Cl^−^ and generate much larger photocurrents (approx. 1000-fold) than those of Arch^18^. Such the excellent light sensitivity of GtACRs has been even more enhanced by soma-targeting in neurons of a mouse brain^19^. GtACRs are thus expected to be more favorable for “efficiently” silencing neural activities “without pH change” in living animals (Fig. 1). In fact, GtACRs have already been proven to function as efficient neural inhibitors in *Drosophila*^20,21^, larval zebrafish^22^, ferret^23^ and mouse^19^. In addition, very recently GtACRs were expressed in “muscle” cells of *C*. *elegans* to control the behavior^24^. Moreover, GtACRs were successfully applied into the neurons of *C*. *elegans* to elucidate the functions of motor neurons in neural circuits^25,26^.

We report here a quantitative *in vivo* demonstration of optical neural silencing of GtACRs in *C*. *elegans* and their applicability for long-term light illumination. Demonstrating the utility of GtACRs in *C*. *elegans* “neurons” is very fruitful for researchers because *C*. *elegans* is a suitable animal model for investigating the mechanism of neural circuits due to its following advantages: (i) a small transparent body, and (ii) a compact and well-characterized nervous system consisting of 302 neurons^27^. We generated transgenic worms expressing GtACR1 or GtACR2 in neurons and quantitatively examined how efficiently those GtACRs can control the behavior of freely moving *C*. *elegans*. The results demonstrate that the light intensity required for GtACRs (approx. 1 µW/mm^2^) to sufficiently cause locomotion paralysis was three orders of magnitude smaller than that required for Arch (approx. 1 mW/mm^2^). We also monitored the lifespan of *C*. *elegans* and the stable silencing effect on orexin neurons in the hypothalamus of mice under long-term illumination. These results prove that GtACRs can be a useful tool for optogenetics in terms of sensitivity, harmlessness and sustainability.

## Results and Discussion

### Functional expression of GtACR1 and GtACR2 in *C*. *elegans* neurons

**Figure 2:**
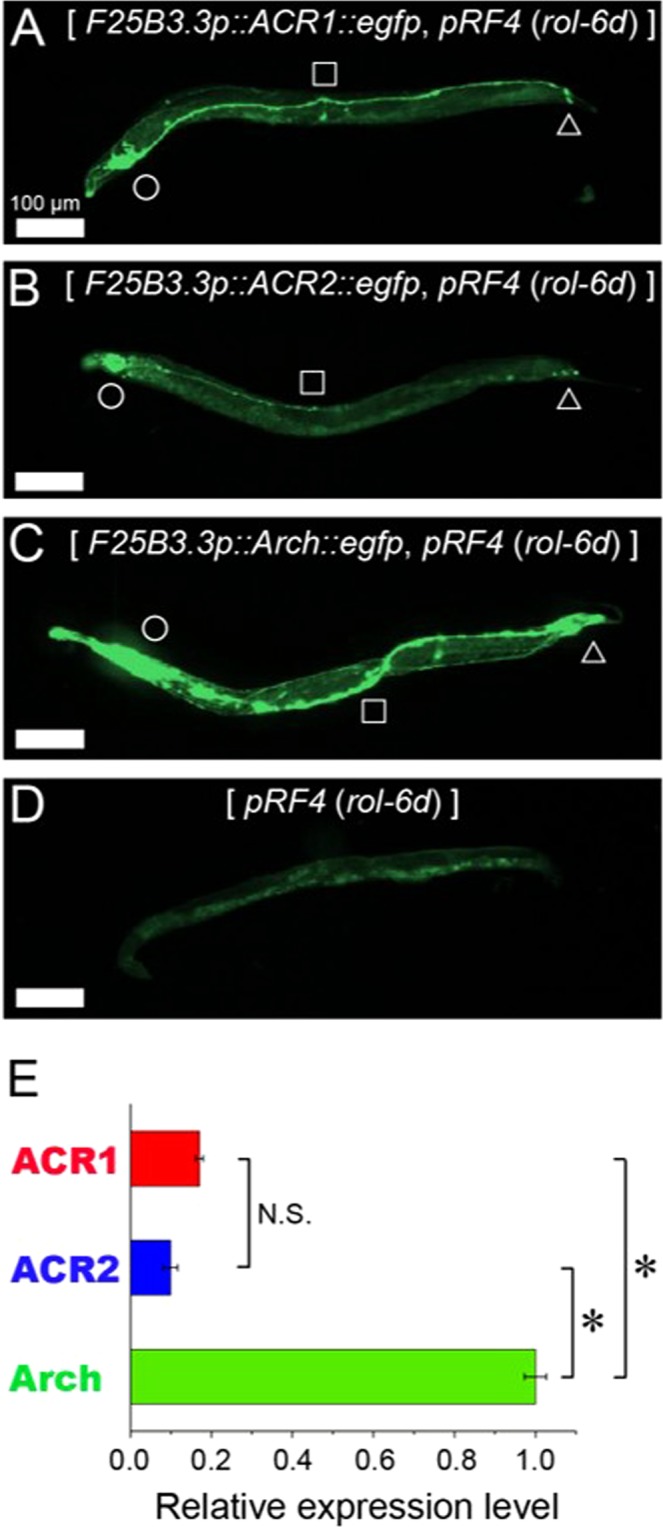
Fluorescence images of worms with plasmids encoding ACR1::eGFP, ACR2::eGFP or Arch::eGFP and their quantitative analysis. (**A–D**) Fluorescence images of worms with plasmids encoding ACR1::eGFP (**A**), ACR2::eGFP (**B**) or Arch::eGFP (**C**) or without rhodopsin (**D**). The pRF4 plasmid (*rol-6d*) was injected into the worms as a transgenic marker conferring the Roller phenotype. Each position of head, ventral nerve cord and tail are respectively depicted by circles, squares and triangles. All images were acquired through a ×10 objective lens and an excitation (460–480 nm)/emission (495–540 nm) filter. The light intensity on the sample and the camera exposure time were 2.8 mW/mm^2^ and 100 ms, respectively. Scale bars indicate 100 µm. (**E**) Quantitative estimation of the total eGFP-fluorescence signals. The results for 5 animals were averaged and are shown with standard error (SE) bars. Asterisks and “N.S.” indicate a significant difference (*p < 0.001; Tukey’s test) and “no significant difference”, respectively.

To confirm the protein expression levels of GtACR1 and GtACR2 in *C*. *elegans*, the gene for green fluorescent protein (eGFP) was fused to the C-termini of GtACR1 and GtACR2 (Fig. S1). The eGFP-tagged GtACR1 (ACR1-eGFP), GtACR2 (ACR2-eGFP) and Arch (Arch-eGFP) were expressed in worms using the promoter of the *F25B3*.*3* gene, which drives pan-neuronal expression. Fluorescence images of ACR1-eGFP (Fig. 2A), ACR2-eGFP (Fig. 2B) and Arch-eGFP (Fig. 2C) in *C*. *elegans* were obtained using a fluorescence microscope with appropriate excitation and emission wavelengths (460–480 nm and 495–540 nm, respectively). A fluorescence image of *C*. *elegans* lacking the rhodopsin gene was also captured to monitor autofluorescence (Fig. 2D). The green fluorescence signals in Fig. 2A–C were significantly stronger than the autofluorescence signal in Fig. 2D and were assigned based on the literature^27^ to be from the head ganglia (circles), ventral nerve cord (squares) and tail ganglia (triangles), indicating the successful expression of ACR1-eGFP, ACR2-eGFP and Arch-eGFP in neurons throughout the body. A linear relationship between the fluorescence intensity and the camera exposure time was ensured to correctly perform quantitative evaluation of the fluorescence signals from eGFP for GtACR1, GtACR2 and Arch (R = 0.99) (Fig. S2). Quantitation of the fluorescence signals (Fig. 2E) revealed that the estimated expression levels of GtACR1 and GtACR2 were approximately 6 and 10 times smaller than that of Arch, respectively. It can thus be concluded that the opsins for GtACRs were expressed in *C*. *elegans* neurons although the expression levels were significantly lower than the expression level of Arch. The low expression levels of GtACRs may be attributed to potential issues concerning transcription, folding and/or stability.

We next examined whether light illumination could affect the locomotion behavior of transgenic worms expressing GtACRs. Since the absorption maxima were reported to be 515 nm for GtACR1^18^, 470 nm for GtACR2^18^ and 552 nm for Arch^28^, neural silencing activity was monitored at wavelengths of 520 ± 10 nm for GtACR1, 460 ± 10 nm for GtACR2 and 545 ± 10 nm for Arch. Upon illumination, the locomotion of worms expressing GtACR1 or GtACR2 were paralyzed immediately, as previously reported for Arch^14^, and locomotion rapidly recovered to normal within 1.0 sec after turning off the light (Supplementary Video 1). In contrast, transgenic worms expressing GtACR1 or GtACR2 opsins without all-*trans* retinal (ATR), an essential cofactor for rhodopsin, showed no locomotion paralysis regardless of the presence of illumination (Supplementary Video 2). These results suggest that, in the presence of ATR, GtACR1 and GtACR2 are functionally expressed in *C*. *elegans* neurons and effectively silence motor neurons governing contraction and relaxation of body wall muscles as previously demonstrated for Arch^14^. Although it is unclear whether the worms possess endogenous retinal and/or its related compounds, even if they exist, the amount(s) must be insufficient for activation of GtACRs. Thus, these results proved that the paralysis elicited in the presence of ATR was indeed caused by GtACRs similar to the proton pump Arch.

**Figure 3:**
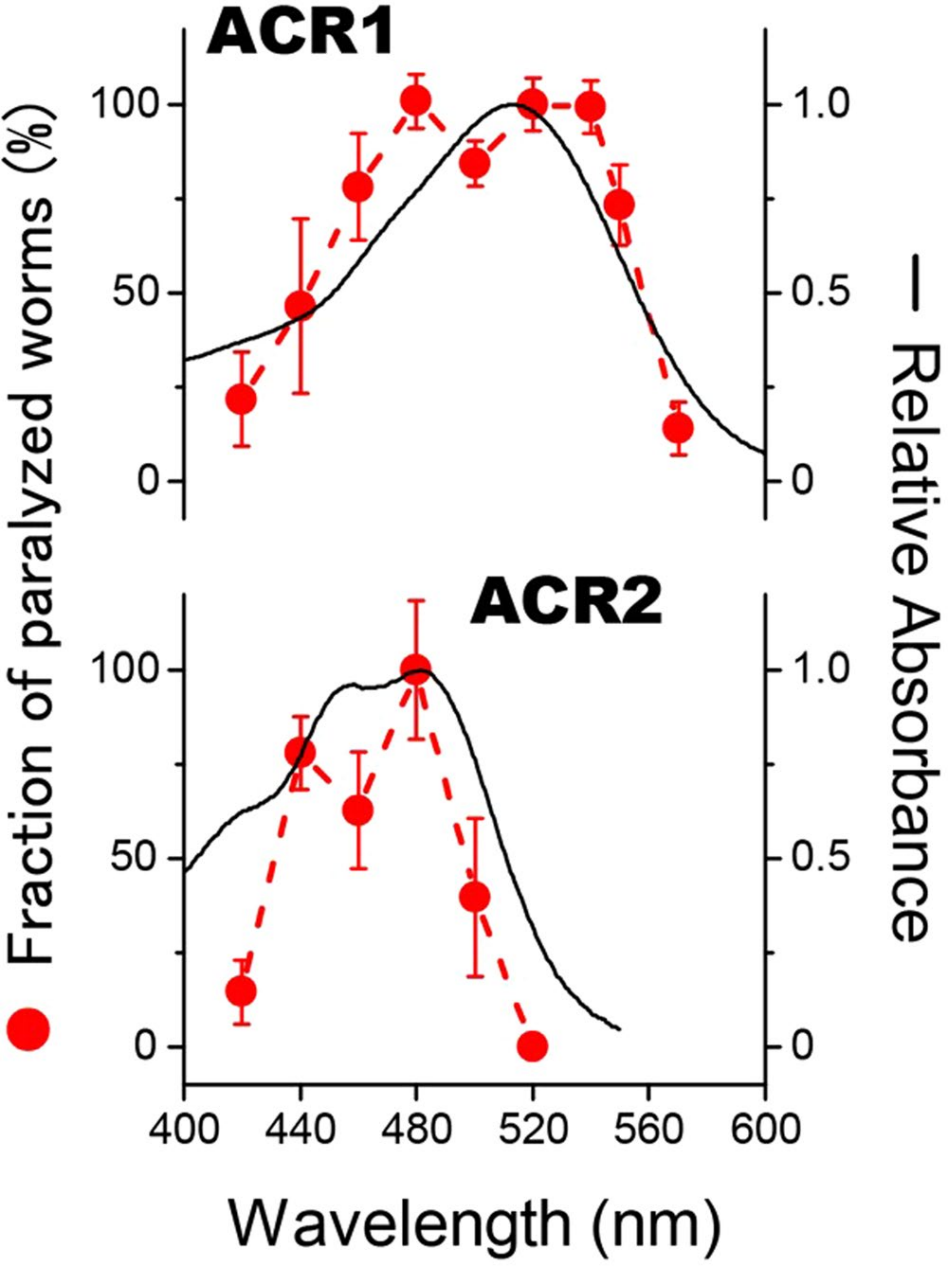
Relationship between the fraction of paralyzed worms expressing GtACRs and wavelengths of light. Fractions of paralyzed worms expressing GtACR1 or GtACR2 were plotted at varying wavelengths of light (red circles). The light intensity for all wavelengths was adjusted to 12 × 10^−4^ mW/mm^2^ for GtACR1 and to 16 × 10^−4^ mW/mm^2^ for GtACR2. The relative absorbance of GtACR1 and GtACR2 purified from HEK293T cells are shown as black lines for comparison. The results for 15 transgenic worms were averaged and are shown with standard error (SE) bars.

Next, to further confirm whether these responses are attributable to GtACRs, we measured the fraction of paralyzed worms against the excitation light wavelengths (Fig. 3). Linear fittings could be performed with the plots when light intensities were below 12 × 10^−4^ mW/mm^2^ for GtACR1 and below 16 × 10^−4^ mW/mm^2^ for GtACR2 (Fig. S3), and therefore we employed these ranges of intensity for the measurements. The absorption spectra of purified GtACR1 and GtACR2 were obtained using HEK293T cells in culture and are shown in Fig. 3 for comparison. The absorption maxima were located at 515 nm and 480 nm for GtACR1 and GtACR2, respectively, and fit with the absorption spectra obtained by electrophysiological experiments in HEK293T cells as reported previously^18^, suggesting the successful purification of GtACRs from HEK293T cells. Of note, this is the first absorption spectrum for purified GtACR2. The fractions of paralyzed worms expressing GtACR1 and GtACR2 roughly matched with the cognate absorption spectra (Fig. 3). However, for GtACR1, the fraction of paralyzed worms showed the slight increase at around 460 nm in comparison with the absorption spectrum. The explanation for this could be that some photointermediate(s) such as an L intermediate is excited to directly return to original state upon the illumination at 460 nm and this shortened photocycle contributes to increase the fraction of paralyzed worms at 460 nm^29^. Similarly, for GtACR2, the absorption spectrum at less than 440 nm seems to be slightly larger than the fraction of paralyzed worms. We presume that this was caused by the impurity in GtACR2 protein purified from HEK293T cells. Importantly, the overall match between the fraction curves and the absorption spectra for both GtACR1 and GtACR2 indicates that GtACR1 and GtACR2 are functionally expressed in *C*. *elegans* neurons and work as an *in vivo* neural silencer.

### Quantitative evaluation of the neural silencing activity of GtACRs

**Figure 4:**
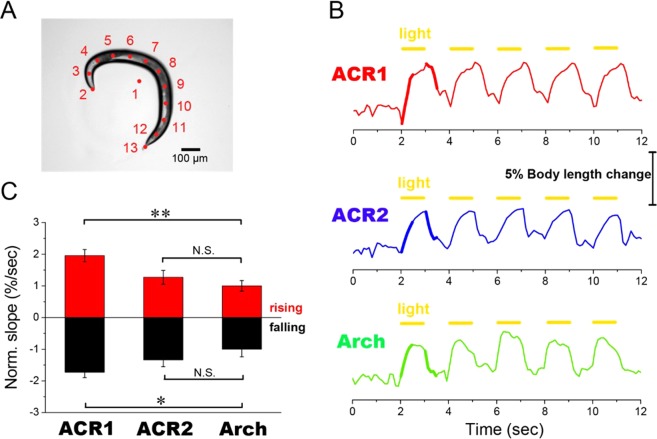
Changes in body length of worms expressing GtACR1, GtACR2 or Arch upon illumination. (**A**) The total body length of each worm was calculated using the software Move-Tr/2D by which its whole body was divided into 11 segments by placing 12 dots (#2~#13) at equal intervals and the distance between dots was then summed. The centroid of the body is shown as dot #1. The scale bar represents 100 µm. (**B**) Changes in body length were induced using 5 light pulses with a 1.0 sec duration at 1.0 Hz frequency. Traces of body length changes were averaged with 5 different worms. The intensities and wavelengths of the light were 3.2 × 10^−3^ mW/mm^2^ and 520 ± 10 nm for GtACR1, 9.0 × 10^−3^ mW/mm^2^ and 460 ± 10 nm for GtACR2 and 4.7 mW/mm^2^ and 545 ± 10 nm for Arch, respectively. These values were enough for activation of GtACRs and Arch. (**C**) The normalized slope of body length change per sec was evaluated from rising and falling curves shown in panel-B after turning the light on and off. The results for 5 different worms were averaged and are shown with standard error (SE) bars. Asterisks and “N.S.” indicate significant differences (^*^p < 0.05, ^**^p < 0.01; Tukey’s test) and “no significant difference”, respectively.

In order to quantitatively evaluate the neural silencing activities of GtACR1 and GtACR2 and to compare them with Arch, we measured the body length changes of worms with or without illumination. As shown in Fig. 4A, we defined the body length of each worm as the sum of the length of each line connecting adjacent dots placed at equal intervals along the midline of the body image of a worm, as reported previously^30^. To investigate body length changes, 5 successive light pulses of 1.0 sec duration and 1.0 Hz frequency were applied to each transgenic worm. As shown in Fig. 4B, in transgenic worms expressing GtACR1, GtACR2 or Arch, elongation of the body length was induced by approximately 5% immediately by the illumination. That elongation was likely to be mediated by the silencing of motor neurons governing muscular contraction^16,30^. After turning off the light, the body length rapidly recovered to the initial level within 1.0 sec. To quantitatively evaluate the response and recovery times, we calculated the slopes of the rising and falling curves for GtACR1, GtACR2 and Arch as represented by the bold lines. As seen in Fig. 4C, the normalized slope of body length change per sec for GtACR2 was comparable to that of Arch, suggesting that GtACR2 efficiently induces neural silencing in freely moving *C*. *elegans*. On the other hand, the normalized slope of body length change per sec for GtACR1 was significantly larger than that for Arch. Since the slope of body length change would reflect the time constant of photocurrents induced by rhodopsins, such the large slope for GtACR1 suggests the ability of the high temporal neural silencing for GtACR1.

**Figure 5:**
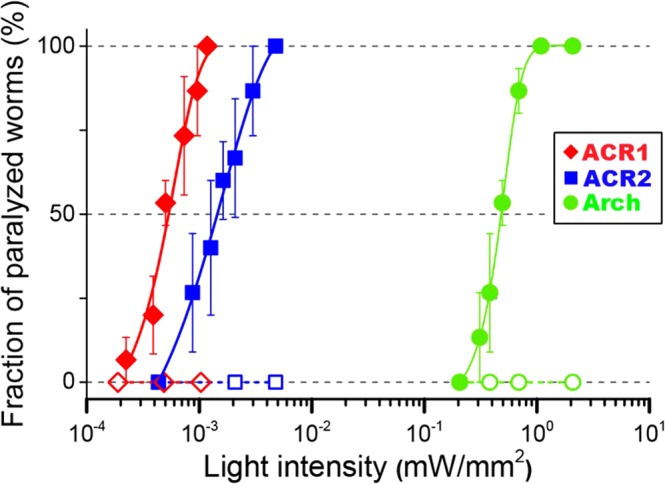
Comparative evaluation of the efficiency of locomotion paralysis by GtACR1, GtACR2 or Arch. The fraction of paralyzed worms expressing GtACR1 (closed diamonds), GtACR2 (closed squares) or Arch (closed circles) was plotted at varying light intensities. The results for 15 transgenic worms were averaged at each light intensity and are shown with standard error (SE) bars. Five worms expressing the pRF4 plasmid (*rol-6d*) showing a Roller phenotype (*ie*., without rhodopsin) accumulated by different light wavelengths (460 nm; open squares, 520 nm; open diamonds, 545 nm; open circles) are shown as negative controls. The light intensity for 50% locomotion paralysis (EI_50_) was estimated by fitting each plot with a Boltzmann distribution.

To further examine the efficiency of GtACRs as a tool for neural silencing, we evaluated and compared the light intensity-dependence of neural silencing activities among GtACR1, GtACR2 and Arch. As shown in Fig. 5, the fraction of paralyzed worms increased as expected in a light intensity-dependent manner for GtACR1 with 520 nm light (closed diamonds), GtACR2 with 460 nm light (closed squares) and Arch with 545 nm light (closed circles). In contrast, worms without any rhodopsin showed no paralysis at all even with increased light intensity or different light wavelengths (460 nm; open squares, 520 nm; open diamonds, 545 nm; open circles). Lastly, we estimated the light intensity for 50% locomotion paralysis (EI_50_). The EI_50_ values for GtACR1, GtACR2 and Arch were estimated to be 5.3 × 10^−4^ mW/mm^2^, 15 × 10^−4^ mW/mm^2^ and 4.8 × 10^−1^ mW/mm^2^, respectively. Thus, GtACR1 and GtACR2 efficiently induced locomotion paralysis of the worms at a roughly 3 orders of magnitude lower light intensity compared with Arch. These results demonstrate that GtACRs have ultra-efficient neural silencing activities in *C*. *elegans*. The reason for such high activities is because GtACRs conduct large number of ions during photoreactions unlike Arch, resulting in drastic changes in the membrane potential. Of note, as shown in Fig. 2, the protein expression levels of GtACRs were 6 to 10-fold smaller than that for Arch in *C*. *elegans*. Assuming that the expression level is linearly correlated with the sensitivity for neural silencing, GtACRs would show 3,000 to 5,000-fold larger sensitivity than Arch. In addition, GtACR1 and GtACR2 would be useful as *in vivo* green and blue-light sensitive neural silencers because of the difference in absorption spectrum. Thus, instead of Arch, GtACRs would be next generation tools for *in vivo* optogenetics with high frequency and ultra-high sensitive neural silencing.

### *In vivo* applicability of GtACRs under long-term illumination

**Figure 6:**
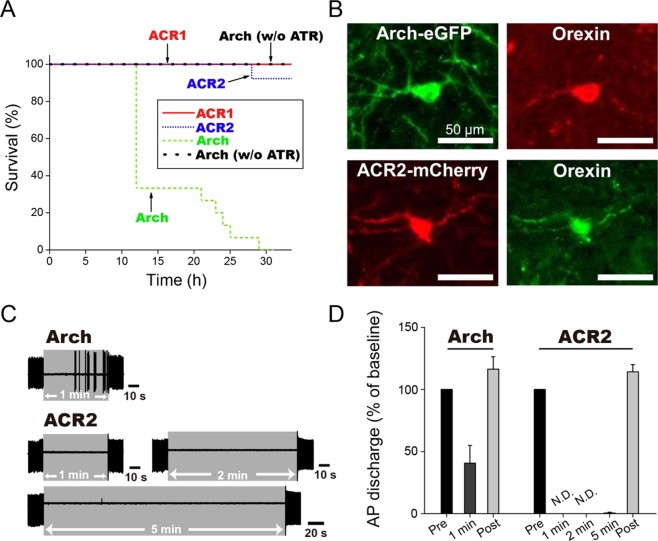
Lifespans of worms under long-term illumination and continuous neural silencing activity in orexin neurons. (**A**) The lifespans of worms expressing Arch or GtACRs under long-term illumination. Fifteen Day1 adult worms expressing Arch or GtACRs with ATR were used, while 15 worms expressing Arch opsin without ATR were used as a control (Arch w/o ATR). The worms were accumulated by light of 545 ± 10 nm for Arch with or without ATR, 520 ± 10 nm for GtACR1 and 460 ± 10 nm for GtACR2. The light intensities were adjusted to 2.8 mW/mm^2^ for Arch, 3.0 mW/mm^2^ for Arch without ATR, 2.1 × 10^−3^ mW/mm^2^ for GtACR1 and 6.6 × 10^−3^ mW/mm^2^ for GtACR2 to sufficiently silence neurons (see Fig. 5). When a worm failed to respond to a gentle touch with a worm picker, it was interpreted as a dead worm. (**B**) Fluorescence images of orexin neurons expressing Arch (upper panels) or GtACR2 (lower panels). The expression of Arch or GtACR2 was confirmed by tagging them with fluorescence proteins, eGFP and mCherry, respectively. Orexin neurons were identified by immunostaining with an anti-orexin antibody (Orexin-A Antibody (C-19), Santa Cruz Biotechnology, USA) and a secondary antibody labeled with fluorescent dyes (CF®594 donkey anti-goat IgG for Arch and CF®488 A donkey anti-goat IgG for GtACR2, Biotium, USA). Scale bars in all images indicate 50 µm. (**C**) Action potential in orexin neurons expressing Arch or GtACR2 during continuous illumination. The light intensity and wavelength were 0.1 mW/mm^2^ and 549 ± 7.5 nm for Arch, and 0.1 mW/mm^2^ and 475 ± 17.5 nm for GtACR2, respectively. (**D**) Quantitative evaluation of firing frequency of action potential (AP) during the continuous illumination. Error bars and “N.D.” represent standard error (SE) and “not detected”, respectively.

As described above and as shown in Fig. 1, the proton pumping rhodopsin Arch causes an unfavorable enrichment of proton concentration (*i*.*e*., acidification) outside the cell. Assuming that an unfavorable proton enrichment induces undesired harmful effects on cells, especially under long-term illumination, we measured the lifespan of worms expressing Arch by observing their motility. Figure 6A shows that worms expressing Arch were completely dead within 30 h under green (545 ± 10 nm) light illumination, where the light intensity was adjusted to be 2.8 mW/mm^2^ to fully activate Arch (see Fig. 5). Although the survival rate of the worms starts changing after 12 h, there should have been already some influence on the neurons of worms even within 12 h. Indeed, it was observed that the body gradually got black and also the motility decreased within 12 h. Similar experiments were also performed for Arch opsin without ATR. Over more than 30 h, the worms survived and their body color and motility hardly changed. These results suggest that the proton transport by Arch causes the decreased lifespan of the worms. Although the physiological reason why the worms with Arch showed decreased lifespan is still unclear, we hypothesize that the extracellular pH change caused by Arch may induce apoptosis, a type of cell death, to neurons via activation of acid-sensing cation channels (ASICs). It has been reported that proton enrichment outside cells activates ASICs^31^. The activated ASICs conduct Ca^2+^ ions and it has been indicated that enrichment of the Ca^2+^ concentration inside cells triggers mitochondria-dependent apoptosis^32^. Demonstrating the above hypothesis will be our next plan. On the other hand, GtACRs conduct Cl^−^ ions according to its concentration gradient from outside to inside across the membrane. Such ion transport naturally occurs in living cells (Fig. 1), and therefore harmlessness of GtACRs can be expected. In fact, worms expressing GtACR1 or GtACR2 could survive for 30 h under green (520 ± 10 nm) light at an intensity of 2.1 × 10^−3^ mW/mm^2^ for GtACR1 and blue (460 ± 10 nm) light at an intensity of 6.6 × 10^−3^ mW/mm^2^ for GtACR2. Those light intensities were sufficient for the full activation of GtACRs (see Fig. 5). To check whether the inhibitory effects of GtACRs were sustained in *C*. *elegans* during long-time illumination, we observed the locomotion of *C*. *elegans* every two hours under microscope. The worms expressing GtACRs have showed almost no movement within two hours from the onset of light illumination and thus GtACRs should have continuously inhibited the neurons of the worms. The body color and the motility of worms did not change within 30 h. Thus, GtACRs can be useful for long-term illumination with harmlessness.

To further clarify the additional side effects of Arch, we measured continuous neural silencing activity upon illumination. Arch was expressed in orexin neurons in the hypothalamus of mice, which has been implicated in the regulation of sleep/wakefulness and was reported to have spontaneous firings (1-2 Hz) in brain slice conditions^33^. Orexin neurons were loose–cell attached to record spontaneous firing events. As shown in the upper panels of Fig. 6B, the expression of Arch in cultured orexin neurons was successfully confirmed by green fluorescence from eGFP (left panel). The presence of the orexin neuron was proven by immunostaining with the fluorescent dye CF®594 for Arch (right panel). Panels C and D in Fig. 6 show spontaneous firing events and quantitative evaluation of their frequency during the continuous illumination. As seen, orexin neurons with Arch started firing even during 1 min illumination, presumably due to acidification outside the cells. We hypothesized that the ion transport function of ion transporter(s) naturally expressed in neurons is altered upon H^+^-induced hyperpolarization by Arch, resulting in the misfiring of the neurons. For comparison, we also expressed GtACR2 in orexin neurons as shown in the lower panels of Fig. 6B. The expression of GtACR2 was successfully confirmed by the red fluorescence from mCherry (left panel), while the presence of orexin neurons was proven by staining with the fluorescent dye CF®488 A (right panel). As seen in Fig. 6C,D, GtACR2 completely inhibited spontaneous firing up to at least 5 min. These results proved that GtACR2 works as a stable neural silencer for a long period without any undesired firing.

In conclusion, we demonstrated in this study that GtACRs can be an *in vivo* powerful neural silencing tool in optogenetics providing extremely high sensitivity, high temporal resolution, harmlessness and long-term silencing. We believe that the advantages of GtACRs would be of high interests for neuroscientists.

## Methods

### Plasmid construction for transgenic *C*. *elegans*

The expression plasmid for Arch was prepared as a fusion construct with the gene encoding fluorescent protein eGFP as described previously^14^. Expression plasmids for ACR1-eGFP and ACR2-eGFP were prepared according to the construction strategy shown in Fig. S1. In short, genes encoding the seven-transmembrane (TM) domains of GtACR1 (Genbank accession no. KP171708, amino acid residues from the 1st to the 269th position) and GtACR2 (Genbank accession no. KP171709, amino acid residues from the 1st to the 266th position), which are sufficient for anion transport activity^34^, were chemically synthesized by GenScript Japan Inc (Tokyo, Japan) with NdeI and XhoI restriction enzyme sites as described previously^34^. Those genes were then inserted into the pET22b plasmid vector using the NdeI and XhoI restriction enzyme sites. During the process, the plasmid pDEST-*F25B3*.*3p* (a kind gift from Dr. Hidehito Kuroyanagi)^35,36^ was used as a destination vector. Finally the genes were fused with eGFP on the expression plasmid using the Gateway system (Invitrogen, USA). This cloning strategy resulted in the following peptide sequences: ACR1[^1^MSSI----NVDG^269^]-TS-eGFP and ACR2[^1^MASQ----KPEA^266^]-TS-eGFP (Fig. S1). The underlined letters indicate the additional restriction enzyme site SpeI, which was used for the gene replacement among genes for Arch, GtACR1 and GtACR2. All constructed plasmids were analyzed using an automated sequencer (ABI 3500 Genetic Analyzer, Life Technologies Ltd., USA and Eurofins Genomics, Japan) to confirm the expected nucleotide sequences.

Transgenic *C*. *elegans* worms were generated by microinjection of the plasmid DNAs (150 ng) into the distal arms of gonads of N2 hermaphrodites as previously described^14,30^. In addition to those plasmids, the pRF4 plasmid (*rol-6d*, 150 ng), a transgenic marker conferring the Roller phenotype, was injected together into the worms to create the transgenic line *Ex(F25B3*.*3p::ACR1::egfp*, *rol-6d)*, *Ex(F25B3*.*3p::ACR2::egfp*, *rol-6d) and Ex(F25B3*.*3p::Arch::egfp*, *rol-6d*). Worms were grown in the dark on Nematode Growth Medium (NGM) plates seeded with a solution of *Escherichia coli* OP50 and 500 µM ATR (Sigma, USA) as described previously^14,30^.

### Microscopic observation

The fluorescence signals for eGFP were observed using an IX71 inverted microscope (Olympus, Tokyo, Japan) with a fluorescence mirror unit (U-MGFPHQ, Olympus) and a mercury lamp (U-LH100HGAPO, Olympus). Fluorescence images were acquired with the CCD camera (ORCA-AG, Hamamatsu Photonics, Japan) using MetaVue software (Molecular Devices, USA) and were analyzed using ImageJ (NIH, Bethesda, MD, USA) and MATLAB (MathWorks, USA) software. The expression levels of the opsins were estimated by subtracting the sum of the autofluorescence in Fig. 2D from the sum of the fluorescence in Fig. 2A–C as expressed in the following equation (1);

1 $${P}_{{eGFP}}^{K}=\sum _{i=1,j=1}^{M,N}{I}_{i,j}^{K}-\sum _{i=1,j=1}^{M,N}{I}_{i,j}^{D},$$

where ${I}_{i,j}^{K}$ is the pixel value of the fluorescence image at (*i*, *j*) pixel and the symbol *K* indicates the image 2 A, 2B or 2 C, *M* and *N* are respectively the number of pixels along the *x*- and *y*-axes, and ${P}_{{eGFP}}^{K}$ is the total eGFP fluorescence signal as the expression level.

For locomotion analysis under varying light intensities, 15 transgenic worms were individually transferred to a fresh NGM plate containing 500 µM ATR, where worms exhibiting the Roller phenotype were chosen as candidate transgenic worms. With strains in which the expression of ACR1-eGFP or ACR2-eGFP was driven by a pan-neuronal promoter, worms showing intense eGFP signals were further selected among Rollers for the assay. The worms were illuminated with a mercury lamp through cognate excitation filters, MX0520 (520 ± 10 nm, Asahi Spectra, Japan), MX0460 (460 ± 10 nm, Asahi Spectra), and U-MRFPHQ (545 ± 10 nm, Olympus) for ACR1-eGFP, ACR2-eGFP and Arch-eGFP, respectively. Images of moving animals were recorded using a CCD camera mounted on the microscope. The relative body length of each worm before and after the illumination was also calculated using image processing and analysis software, Wriggle Tracker (Library Co., Japan) and Move-Tr/2D (Library Co.). The light pulses for inducing the body length change were constructed by an optical shutter (VMM-T1J, VINCENT ASSOCIATES, USA). For measurements of the fraction of paralyzed worms at specific wavelengths and intensities of light, we used 9 band-pass filters (full width at half-maximum (FWHM) = 10 nm, Asahi Spectra) and 6 neutral-density (ND) filters (OptoSigma, Japan). The light intensity was measured at the object plane using an optical power meter (#3664, Hioki, Japan) with an optical sensor (#9742, Hioki). Whether the worms showed paralysis or not was judged by observing its whole body movement within 1.0 second after the onset of the illumination. For the series of illumination experiments with varying light intensities (from 1.9 × 10^−4^ to 3.0 mW/mm^2^), worms were used only once, and were then discarded. All experiments were performed at room temperature (20–25°C) in the dark.

### Expression and purification of GtACRs expressed in HEK293T cells

The cDNAs encoding the 7 TM domains of ACR1 (from the 1st to the 295th position) and ACR2 (from the 1st to the 291th position) were fused with a hexahistidine-tag sequence at their C-termini and were inserted into the mammalian expression vector pCAGGS as previously described^37,38^. The plasmids were transfected into HEK293T cells using the calcium-phosphate method and protein expression was carried out according to previously described methods^37,39^. After a one day incubation of the transfected cells, ATR was added into the medium (final concentration; 5 μM). After an additional day incubation in the dark, the cells were collected by centrifugation (7,500 × g, 10 min) at 4 °C. The collected cells were disrupted by sonication and solubilized in Buffer A (50 mM HEPES and 140 mM NaCl, pH 6.5) containing 1% w/v n-dodecyl-β-D-maltoside (DDM, Dojindo Laboratories, Japan). The solubilized fraction was collected by ultracentrifugation (123,400 × g, 30 min) at 4 °C and the supernatant was applied to a Ni^2+^ affinity column (GE Healthcare, USA). Thereafter, the column was washed extensively with Buffer B (20 mM HEPES, 300 mM NaCl, 5% w/v glycerol, 0.02% DDM, pH 7.4) containing 20 mM imidazole to remove nonspecifically bound proteins, after which the histidine-tagged proteins were eluted with a linear gradient of imidazole. For GtACR1, the purified proteins were then further applied to an anion-exchange column (GE Healthcare) in Buffer C (20 mM Tris, 0.05% DDM, pH 8.0). Thereafter, the column was washed extensively with Buffer C and then eluted with a linear gradient of NaCl. The purified proteins were concentrated and their buffers were exchanged using an Amicon Ultra filter (30,000 Mw cut-off; Millipore, USA) to Buffer B. Absorption spectra of the purified GtACRs were recorded using an UV-visible spectrophotometer (Shimadzu UV-2400, Japan) at room temperature (around 25 °C).

### Animal usage

All experimental protocols that involved animals in this project were approved by the Institutional Animal Care and Use Committees, Research Institute of Environmental Medicine, Nagoya University, Japan. All experiments in this study involving the use of mice were performed in accordance with approved guidelines of the Institutional Animal Care and Use Committees, Research Institute of Environmental Medicine, Nagoya University, Japan. All efforts were made to reduce the number of animals used and also to minimize the suffering and pain of animals. Animals were maintained on a 12-hour light-dark cycle (lights were turned on at 8:00 am), with free access to food and water.

### Electrophysiological measurements of murine orexin neurons

Acute brain slice preparations and subsequent electrophysiological experiments were adopted from a protocol published elsewhere^40^. Briefly, adult male mice (4–5 months old) with a genotype of either *orexin-EGFP-Cre* or *orexin-Arch-GFP*, in which orexin-producing neurons (orexin neurons) exclusively express Cre recombinase or Arch under control of the prepro-orexin promoter, respectively, were used for electrophysiological recordings. A Cre-inducible adeno-associated virus (AAV) vector carrying GtACR2 (AAVdj-CMV-FLEX-ACR2-2A-mCherry) was injected into the lateral hypothalamus (600 nL, 1 × 10^13^ particles/ml) of *orexin-EGFP-Cre* mice. Three weeks post-injection, mice were anesthetized and decapitated, after which their brains were quickly removed and chilled in ice-cold cutting solution (in mM: 110 K-gluconate, 15KCl, 0.05 EGTA, 5 HEPES, 26.2 NaHCO_3_, 25 glucose, 3.3 MgCl_2_ and 0.0015 (±)-3-(2-carboxypiperazin-4-yl) propyl-1-phosphonic acid) gassed with 95% O_2_ and 5% CO_2_. Coronal brain slices (300 µm thick) that contained the lateral hypothalamus were generated using a vibratome (VTA-1200S; Leica, Germany). The brain slices were then temporarily preserved in an incubation chamber containing bath solution (124 mM NaCl, 3 mM KCl, 2 mM MgCl_2_, 2 mM CaCl_2_, 1.23 mM NaH_2_PO_4_, 26 mM NaHCO_3_ and 25 mM glucose) and were gassed with 95% O_2_ and 5% CO_2_ in a 35°C water bath. The brain slices were then incubated at room temperature (24–28°C) in the same incubation chamber for 30–60 min. Orexin neurons expressing Arch or GtACR2 in each brain slice were identified by the green fluorescence of eGFP and the red fluorescence of mCherry, respectively. A positive pressure was introduced in the patch pipette and it was moved towards the cell. A loose cell-attached configuration was achieved by making a loose-seal with the cell membrane. Different wavelengths of light were provided by a light source that uses a light-emitting diode (Spectra light engine; Lumencor, USA) and were guided to the BX51WI fluorescent microscope (Olympus) with a liquid light fiber 1 cm in diameter. The brain slices were illuminated with blue (475 ± 17.5 nm, 15.4 mW) or green (549 ± 7.5 nm, 20 mW) light through the objective lens. An Axopatch 200B amplifier (Axon Instruments, Molecular Devices, USA) was used for recording. Data acquisition was performed using pClamp 10.2 software (Molecular Devices) via a Digidata 1322 A (Axon Instruments).

## Supplementary information

Supplementary information

Supplemental Video S1

Supplemental Video S2
