# Supplementary material for: Quantitation of the neural silencing activity of anion channelrhodopsins in Caenorhabditis elegans and their applicability for long-term illumination
Source: Sci Rep. 2019 May 27;9:7863. doi: 10.1038/s41598-019-44308-x (PMC6536681; doi:10.1038/s41598-019-44308-x)
Supplement: Supplementary file 1 — Supplementary information [file 41598_2019_44308_MOESM1_ESM.docx]

**Quantitation of the neural silencing activity of anion channelrhodopsins in *Caenorhabditis elegans* and their applicability for long-term illumination**

Taro Yamanashi^1^, Misayo Maki^1^, Keiichi Kojima^1^, Atsushi Shibukawa^1^, Takashi Tsukamoto^1,#^, Srikanta Chowdhury^2^, Akihiro Yamanaka^2^, Shin Takagi^3^ & Yuki Sudo^1*^

From the ^1^Graduate School of Medicine, Dentistry and Pharmaceutical Sciences, Okayama University, Okayama 700-8530, Japan; ^2^Department of Neuroscience II, Research Institute of Environmental Medicine, Nagoya University, Nagoya 464-8601, Japan; ^3^Division of Biological Science, Graduate School of Science, Nagoya University, Nagoya 464-8602, Japan.

^#^Present address: Faculty of Advanced Life Science and Global Station for Soft Matter, Global Institution for Collaborative Research and Education, Hokkaido University, Kita-10 Nishi-8, Kita-ku, Sapporo 060-0810, Japan.

*To whom correspondence should be addressed: Yuki Sudo: Graduate School of Medicine, Dentistry and Pharmaceutical Sciences, Okayama University, Okayama 700-8530, Japan; [sudo@okayama-u.ac.jp](mailto:sudo@okayama-u.ac.jp); Tel: +81-86-251-7945.

**Supplementary Information**

**
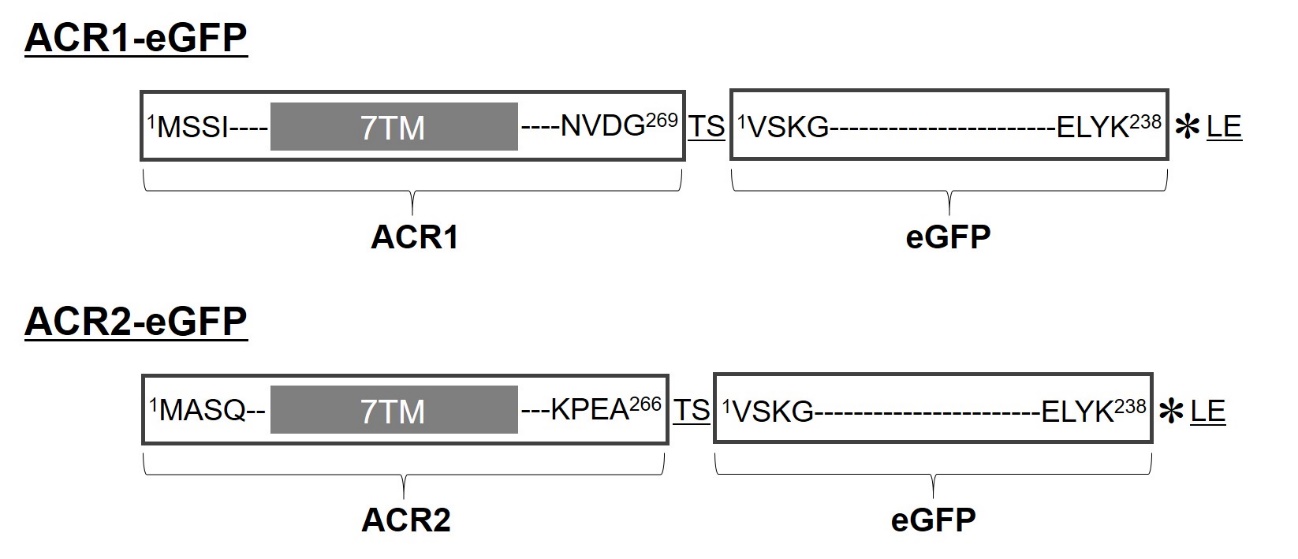
**

**Supplementary Figure S1. Schematic of the genetic sequences of ACR1-eGFP and ACR2-eGFP*.*** GtACR1 (Genbank accession no. KP171708, amino acid residues from the 1st to the 269th position) and GtACR2 (Genbank accession no. KP171709, amino acid residues from the 1st to the 266th position) were C-terminally fused with the eGFP gene (amino acid residues: 1-238) on the plasmid pDEST-*F25B3.3p*. “7TM” indicates the seven-transmembrane domain. TS and LE indicate additional restriction enzyme sites, SpeI and XhoI, respectively. Asterisks represent the three nucleotides encoding the stop codon (TAA).


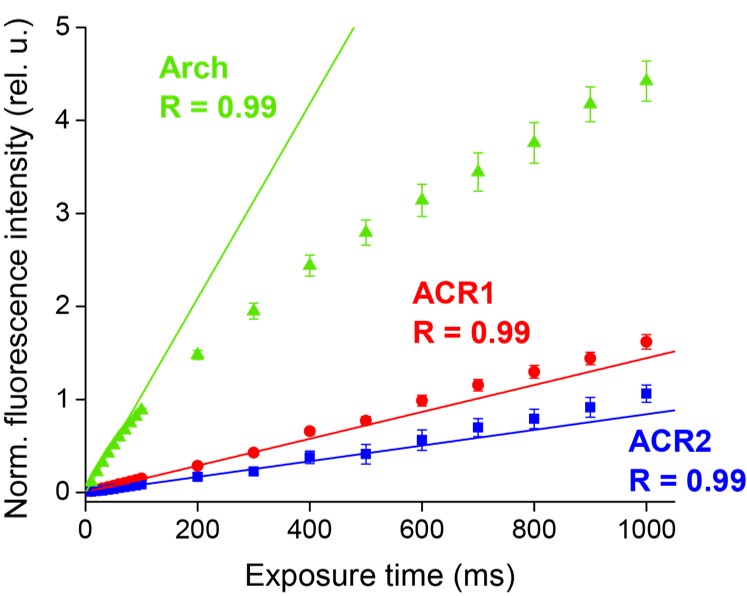


**Supplementary Figure S2. Linearity between fluorescence intensity and camera exposure time for ACR1-eGFP, ACR2-eGFP and Arch-eGFP.** To ensure that the expression levels of GtACRs and Arch are correctly evaluated, the linearity between fluorescence intensity and camera exposure time was measured. The plots were fitted by a linear equation (solid lines) within exposure times up to 300 ms for GtACR1, 200 ms for GtACR2 and 50 ms for Arch. Error bars and “R” indicate standard errors (SE) and the linearity coefficient, respectively.

**
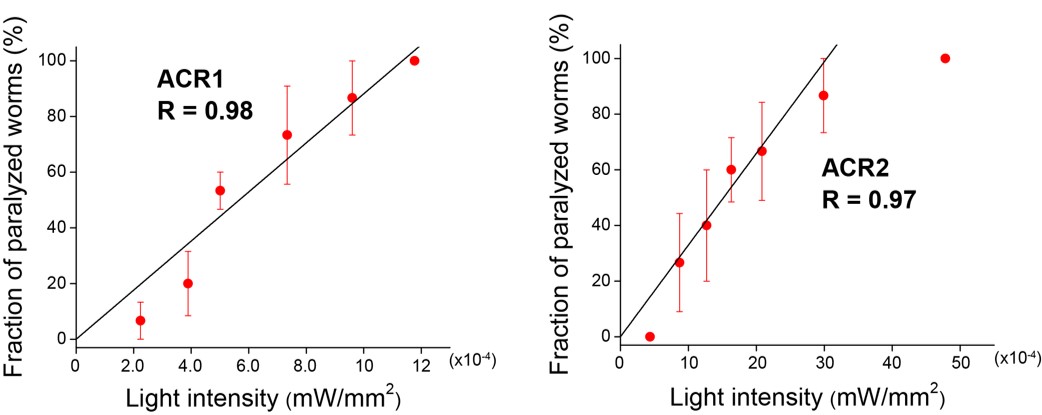
**

**Supplementary Figure S3. Linearity between the fraction of paralyzed worms expressing GtACR1 or GtACR2 and light intensity.** Fractions of paralyzed worms expressing GtACR1 or GtACR2 depending on light intensities were plotted. Linear fittings could be well performed with the plots when light intensities were below 12×10^-4^ mW/mm^2^ for GtACR1 and below 16×10^-4^ mW/mm^2^ for GtACR2. The results for 15 transgenic worms were averaged for each plot. Error bars and “R” indicate standard errors (SE) and the linearity coefficient, respectively.

**Supplementary Video S1. Light-induced changes in locomotion of freely moving *C. elegans* expressing ACR1-eGFP or ACR2-eGFP with ATR*.***

ACR1-eGFP or ACR2-eGFP were expressed in *C. elegans* neurons with ATR (*F25B3.3p::ACR1::egfp, pRF4* (*rol-6d*) and *F25B3.3p::ACR2::egfp, pRF4* (*rol-6d*)). Green (520 ± 10 nm, 2.6×10^-3^ mW/mm^2^) and blue (460 ± 10 nm, 7.1×10^-3^ mW/mm^2^) light pulses for 10 sec were used for the locomotion paralysis of the worm.

**Supplementary Video S2. Locomotion of freely moving *C. elegans* expressing ACR1-eGFP or ACR2-eGFP without ATR.**

ACR1-eGFP or ACR2-eGFP were expressed in *C. elegans* neurons without ATR (*F25B3.3p::ACR1::egfp, pRF4* (*rol-6d*) and *F25B3.3p::ACR2::egfp, pRF4* (*rol-6d*)). Green (520 ± 10 nm, 2.6×10^-3^ mW/mm^2^) and blue (460 ± 10 nm, 7.1×10^-3^ mW/mm^2^) light pulses for 10 sec were applied to the worm.
